# Major Histocompatibility Complex Binding, Eluted Ligands, and Immunogenicity: Benchmark Testing and Predictions

**DOI:** 10.3389/fimmu.2019.03151

**Published:** 2020-02-05

**Authors:** Sinu Paul, Alba Grifoni, Bjoern Peters, Alessandro Sette

**Affiliations:** ^1^Division of Vaccine Discovery, La Jolla Institute for Immunology, La Jolla, CA, United States; ^2^Department of Medicine, University of California, San Diego, San Diego, CA, United States

**Keywords:** anti drug antibodies (ADA), CD4 T cell, MHC-prediction, prediction benchmarking, immunogenicity

## Abstract

Antidrug antibody (ADA) responses impact drug safety, potency, and efficacy. It is generally assumed that ADA responses are associated with human leukocyte antigen (HLA) class II-restricted CD4+ T-cell reactivity. Although this review does not address ADA responses *per se*, the analysis presented here is relevant to the topic, because measuring or predicting CD4+ T-cell reactivity is a common strategy to address ADA and immunogenicity concerns. Because human CD4+ T-cell reactivity relies on the recognition of peptides bound to HLA class II, prediction, or measurement of the capacity of different peptides to bind or be natural ligands of HLA class II is used as a predictor of CD4+ T-cell reactivity and ADA development. Thus, three different interconnected variables are commonly utilized in predicting T-cell reactivity: major histocompatibility complex (MHC) binding, capacity to be generated as natural HLA ligands, and T-cell immunogenicity. To provide the scientific community with guidance in the relative merit of different approaches, it is necessary to clearly define what outcomes are being considered. Thus, the accuracy of HLA binding predictions varies as a function of what the outcome predicted is, whether it is binding itself, natural processing, or T-cell immunogenicity. Furthermore, it is necessary that the accuracy of prediction is based on rigorous benchmarking, grounded by fair, objective, transparent, and experimental criteria. In this review, we provide our perspective on how different variables and methodologies predict each of the various outcomes and point out knowledge gaps and areas to be addressed by further experimental work.

## Introduction

As discussed in general and in more detail in other contributions to this special issue, protein-drug immunogenicity is of concern, as it can lead to safety issues and can impact drug efficacy and potency. It is further widely assumed that immunogenicity at the level of CD4 T cells recognizing human leukocyte antigen (HLA) class II epitopes is a key and necessary step in the development of antidrug antibodies (ADAs), because CD4 T cells are generally required for antibody affinity maturation and isotype switching, which is of relevance because ADA is in general IgG and other subclasses that require immunoglobulin gene rearrangements.

**Figure 1:**
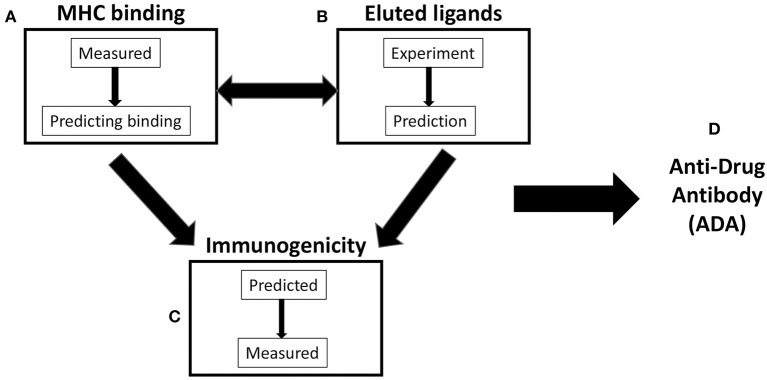
Scheme of three variables commonly considered in antidrug antibody (ADA) prediction. **(A)** Major histocompatibility complex (MHC) binding: MHC-peptide binding can be directly measured and/or utilized to train MHC-binding prediction methods. **(B)** Eluted ligands: naturally processed and presented peptides can be eluted from antigen-presenting cells (APCs) and/or used as a source for the training of algorithm predicting natural ligand generation. **(C)** Immunogenicity: T-cell reactivity is often predicted on the basis of binding or elution data/predictions. In addition, it can be directly measured and/or predicted by methods utilizing T-cell immunogenicity as a training set. **(D)** Antidrug antibody (ADA): it is commonly assumed that ADA responses are dependent on **(A–C)** and related to T-cell reactivity.

As a result, a variety of strategies have been developed to assay and/or predict different steps in the process of the development of ADA. This review will focus on efforts and available data benchmarking different methodologies and outcomes relating to HLA class II binding, elution of natural HLA class II ligands, and T-cell immunogenicity *in vitro*. The interconnection between these different methodologies at the level of actual experimental data vs. bioinformatic prediction is graphically illustrated in Figure 1. This paper is mostly reflective of our work in the context of Immune Epitope Database and Analysis Resource (IEDB), and we fully acknowledge the seminal contributions of several other groups, as also detailed in other contributions to this special issue. Likewise, this review does not address other variables that are appreciated to impact ADA and T-cell immunogenicity, such as induction of T-cell tolerance, self-similarity, protein-drug dosing and schedule, aggregation state, and general immune responsiveness of the drug recipient. We emphasize that the present study is a review, and as such, we do not present primary data presented elsewhere. In each paragraph, the specific papers and sources of the primary data are referenced, to allow the reader a more in-depth analysis if desired.

## Human Leukocyte Antigen Binding and Eluted Ligands

HLA class II binding, more generally major histocompatibility complex (MHC) binding, is measured by *in vitro* utilizing preferably synthetic peptides and purified HLA class II molecules. The most accurate and reproducible “gold standard” assay on hand is a classic radiolabeled probe displacement receptor ligand assay, developed by Gray, Sette, and Buus and Unanue, Babbit, and Allen in the mid-1980s (1, 2). Other assay platforms that have been previously described suffer from difficulties in controlling peptide degradation (live-cell assays) (3) or a low throughput (plasmon resonance assays) (4). Furthermore, radiolabeled probe displacement receptor ligand assay has been run for many different HLA class II allelic variants with a large number of synthetic peptides (5, 6), and it is thus associated with the most numerous volume of accurate and directly comparable data. Accordingly, these data have been used, as described in more detail in the following sections, to train predictive algorithms, which have increased efficacy and accuracy throughout the past three decades (Figure 1A).

Like in the case of all MHC molecules, the vast majority of peptide-binding sites of HLA class II is occupied by natural ligands, derived from antigens processed into small peptides and displayed on the surface of antigen-presenting cells (APCs). These natural ligands can be eluted and characterized (4). In the context of application to the characterization of protein, drug-derived peptides with the acronym MAPPs, which stands for MHC-associated peptide proteomics (MAPPs), are frequently used (7, 8). Recent years have witnessed an explosion of availability of sequences of natural ligands, thanks to the ever-increasing power of mass spectrometry (MS) sequencing techniques (9). As a result, these eluted ligand data can also be used to train predictive algorithms (Figure 1B), as also described in the following sections. It is perhaps intuitively expected that the two different training sets might yield largely overlapping results, with binding data being the most effective in predicting binding capacity and eluted ligand being the most effective to predict eluted ligands but not necessarily HLA binding *per se*.

## T-Cell Immunogenicity

In order for an epitope to be recognized by CD4 T cells, it needs to be capable of binding HLA class II molecules and of being generated by natural processing. Accordingly, binding and natural ligand assays and predictions are routinely utilized to predict T-cell immunogenicity. However, it should be kept in mind that these measures, by definition, do not necessarily relate to immunogenicity at the T-cell level, as other variables are also involved (e.g., the degree of similarity to self-antigens). Furthermore, it is often not clear which thresholds are associated with the optimal prediction of T-cell epitope, on the basis of either measured or predicted elution or binding data.

Alternative strategies use T-cell immunogenicity data to train agnostic predictors or use *in vitro* immunogenicity assays to predict or rank the immunogenicity of protein drugs *in vivo* in humans (Figure 1C). Here as well, considerable challenges and opportunities for further research exist, as it is unclear how specific and sensitive these assays are and how they do correlate with *in vivo* immunogenicity. Likewise, it is unclear whether T-cell immunogenicity *in vitro* in unexposed naïve individuals can predict T-cell immunogenicity in exposed individuals. Finally, and of the greatest relevance, data that demonstrate that T-cell immunogenicity measured by currently used assays does, in fact, correlate with ADA titers in human patient populations are very limited (Figure 1D). Figure 1D is presented here to point out a knowledge gap, and no data for ADA are reviewed herein. Several studies are starting to generate data relevant to this respect, in the context of protein therapeutics that are either human or humanized and foreign proteins such as asparaginase and glucarpidase. These topics are addressed in other papers presented in this issue and are not within the scope of this review. In the context of this paper, we simply point out that the volume of data is as yet insufficient to perform a systematic and unbiased evaluation.

## The Concept and Necessity of Benchmarking Predictive Algorithms

To rigorously evaluate the performance of any predictive algorithm, it is generally necessary to define objective measures of performance. Commonly utilized measures are sensitivity [what fraction of true positives (TPs) are predicted vs. false positive (FP)] and specificity [what fraction of the predictions are TPs vs. false negatives (FN)]. The prediction rates are plotted to generate an area under the curve (AUC) and AUC values, which are an overall numeric assessment of performance (with an AUC of 0.5 being associated with random predictions and an AUC of 1.00 corresponding to a perfect prediction).

Once the method to be used for evaluation is defined, it is necessary to define datasets that are going to be used to assess the algorithm's performance. The evaluation dataset should be distinct from the one used to derive the method, to avoid overfitting. This is particularly the case for heuristic and machine learning approaches, where the method will fit the data without a predefined hypothesis or model. The process by which a different methodology is objectively and rigorously evaluated is generally referred to as “benchmarking.”

In our opinion, to have true scientific value, a benchmarking needs to fit three fundamental characteristics. First, it needs to be objective, following predefined metrics and an accepted methodology. Second, it needs to utilize independent datasets, not used to train the methodology and preferably not available to the method developer while the method was trained. Third, it needs to be transparent, using publicly available code, preferably published in the peer-reviewed literature, and the results must be verifiable and reproducible by anyone in the scientific community.

## Benchmarking Human Leukocyte Antigen Class II Binding Predictions

To the best of our knowledge, the first comprehensive rigorous benchmarking of different prediction methodologies was reported for HLA class I by Peters et al. (2). In those studies, predictions for over 48 MHC alleles, 88 datasets, and 48,828 IC_50_ values were considered, with 50–300 data points per dataset. In general, the performance of different methodologies was similar, and the main factor influencing predictive power was found to be how many data points were available for training predictions for a given allele. Since then, the process of benchmarking was automated and is periodically performed by the IEDB (10).

Following the same thought process and methodologies, we have recently instituted a platform for automated benchmarking of HLA class II predictions (11). On a weekly basis, the absolute and relative predictive performance of all participating tools on data newly entered into the IEDB is assessed before it is made public. This unbiased assessment of available prediction tools is fully automated, and results are posted on a publicly accessible website (http://tools.iedb.org/auto_bench/mhcii/weekly/). The initial benchmarking included six commonly used prediction servers. The results from that process have room for improvement, predictions were reasonably accurate with median AUC values for the various class II molecules of around 0.8 for the best methods (NetMHCIIpan and NNalign). Since the publication of the study, additional gains have been realized with an AUC value of 0.835 for NetMHCIIpan (11). The current benchmarking evaluates MHC binding, and we plan to extend this automated benchmarking to eluted ligand data and eventually T-cell immunogenicity data.

It is important to realize that this benchmarking only assesses the performance on class II binding predictions, in terms of predicting binding itself, and should not be interpreted to assess how well-binding prediction predicts immunogenicity or ADA. Although this would seem self-apparent, we often encounter statements to the extent that the “MHC binding predictions do not work because I have immunogenicity data that ….” Obviously, although the binding is necessary for immunogenicity, it is not the sole condition. The current efforts to objectively assess the performance of HLA class II binding (predicted or measured) as a predictor of HLA class II immunogenicity are described in a section further below.

## Natural Ligand and Processing Predictions

As mentioned above, the recent years witnessed a dramatic increase in the availability of data relating to HLA class II eluted ligands. In this context, a reasonable line of investigation would be to examine if the eluted ligand data could be utilized to learn some “processing motif,” present in natural ligand but not associated with HLA-binding motifs. A recent study by Paul et al. (12) used this approach. MHC II ligand elution data collected from IEDB were further filtered to generate a high-quality dataset. The result was the delineation of a predictive cleavage motif for eluted ligands. A combination of cleavage and binding predictions improved ligand predictions. Strikingly, however, incorporating the processing motif in combination with binding predictions did not improve predictions of which sequences would be actual T-cell epitopes. Similar results were also obtained in a study from Nielsen's group (13), who detected a footprint of antigen processing, which improved predictions of eluted ligands but did not improve predictions of which sequences would be actual T-cell epitopes. These results are remarkably similar to what was previously observed in the case of class I molecules where it was found that processing predictions were not affording increased efficacy in predicting actual T-cell epitopes, either by themselves or in combination with binding predictions.

Previous data by Jurtz et al. (14) demonstrated that directly using eluted ligand data to train neural networks (NNs) was associated with increased capacity to predict eluted ligands, as compared with NN trained in HLA class I binding data. Garde et al. (15) demonstrated that training in class II eluted data increases the accuracy of predicting eluted ligands, just as previously observed in the case of class I. Thus, training NN algorithms with MS eluted ligands improves the capacity to accurately predict eluted ligands for both HLA class I and II alleles (14–16).

## Comparison of Binding and Eluted Ligand Data

In terms of comparing these two different data types, a first question to be addressed is how the measured binding and experimental elution data compare with each other. An analysis performed more than 2 years ago (17) demonstrated that T-cell and MHC-binding data were mostly related to non-self, whereas elution ligands are mostly self. This is largely a reflection of the fact that HLA binding and epitope studies have prevalently been focused on infectious diseases and allergy targets, whereas ligands encountered that are naturally occupying the HLA class II binding site are predominantly of self-origin. Therefore, the problem is just that the particular peptide sets that happen to be studied in the two approaches are non-overlapping, complicating direct comparison but not necessarily leading to different predictions. This is not a reflection of the fact that self and non-self peptides differ in their capacity to bind or to be generated by natural processing. The fact that MHC class II molecules bind indiscriminately the self and non-self peptides were established in the early 1990s (18). The disparity in the number of self vs. non-self peptide data available in the literature and associated with the two techniques is simply a reflection of the investigational bias of MHC-binding and T-cell mapping studies being mostly focused on infectious diseases and allergen targets, whereas in the case of natural MHC ligands, the most abundant species (and therefore more easily sequenced species) are of self-origin. Tables 1, 2 present numbers of peptides eluted from MHC class II molecules. These are the data available through the IEDB as of Q3 2019, which contain the specific peptide sequences and specific MHC class II molecules.

**Table 1:** Composition of epitopes available in IEDB.

		**MHC class II**		
**Classification based on**	**Type**	**T-cell epitopes**	**MHC binders**	**Eluted ligands**
**(A) MHC-BINDING DATA**				
Host	All	62,380	21,885	66,304
	Human	41,577	18,944	53,810
	Rodents/rabbit	21,351	3,644	13,335
	Non-human primates	269	106	NA
	Other hosts	2,181	60	NA
Antigen source	Self	6,251	3,542	63,766
	Non-self	39,983	12,943	1,495
	Viruses	25,834	9,575	484
	Allergen	1,924	1,342	784
	Bacteria	9,358	1,409	151
	Parasites	2,071	553	74
	Fungus	796	64	2
		**Predicted binder or not**		
		**Binder**	**Non-binder**	**Total**
**(B) NATURAL LIGAND ELUTION DATA**				
Eluted or not	Eluted	124	42	166
	Non-eluted	10,397	646,239	656,636
	Total	10,521	646,281	656,802

*IEDB, Immune Epitope Database and Analysis Resource; MHC, major histocompatibility complex*.

**Table 2:** Binding affinity at which 50 and 90% of epitopes are retrieved for each HLA class II allele on the basis of epitope or tetramer data available in IEDB.

	**Epitopes**		**Tetramers**	
	**Affinity at 50%**	**Affinity at 90%**	**Affinity at 50%**	**Affinity at 90%**
DPA1*01:03/DPB1^*^01:01	135	4,220	NA	NA
DPA1^*^01:03/DPB1^*^04:01	104	2,609	NA	NA
DPA1^*^01:03/DPB1^*^04:02	439	3,015	NA	NA
DQA1^*^01:01/DQB1^*^05:01	2,294	9,706	NA	NA
DQA1^*^01:01/DQB1^*^06:02	6,180	1,813	1,141	204
DQA1^*^01:02/DQB1^*^06:02	300	2,439	943	186
DQA1^*^03:01/DQB1^*^03:02	1,091	6,001	NA	NA
DRB1^*^01:01	26	234	171	87
DRB1^*^01:02	553	966	NA	NA
DRB1^*^01:03	NA	NA	3,944.14	1,718.14
DRB1^*^03:01	196	1,823	2,026	7,107
DRB1^*^04:01	204	8,896	146	5,300
DRB1^*^04:03	69	321	NA	NA
DRB1^*^04:04	NA	NA	313.25	225.87
DRB1^*^04:05	1,163	6,678	NA	NA
DRB1^*^04:07	123	696	NA	NA
DRB1^*^07:01	65	565	54	206
DRB1^*^08:01	NA	NA	686.2	1,563.99
DRB1^*^08:02	110	1,673	NA	NA
DRB1^*^08:03	1,009	986	NA	NA
DRB1^*^09:01	66	678	14	194
DRB1^*^10:01	19	46	NA	NA
DRB1^*^11:01	43	582	212	179
DRB1^*^11:04	NA	NA	922.29	145.13
DRB1^*^12:02	119	361	NA	NA
DRB1^*^13:01	44	243	NA	NA
DRB1^*^14:01	NA	NA	380	423
DRB1^*^14:02	423	547	NA	NA
DRB1^*^14:04	222	247	NA	NA
DRB1^*^14:06	47	135	NA	NA
DRB1^*^15:01	115	1,843	382	1,233
DRB1^*^15:06	231	1,528	NA	NA
DRB3^*^02:02	1,233	1,788	NA	NA
DRB5^*^01:01	60	2,811	505	107

*NA, not applicable; HLA, human leukocyte antigen; IEDB, Immune Epitope Database and Analysis Resource*.

Table 1A presents an updated analysis (as of Q3 2019) focused on HLA class II. This analysis highlights how comparing measured HLA binding and eluted data is problematic in general and for HLA class II in particular because the two datasets are only minimally overlapping. This knowledge gap is starting to be addressed by several studies in the context of murine class I molecules (19, 20). Croft et al. utilized the vaccinia virus (VACV) as a model system in the context of the murine MHC class I molecules Kb and Db (19). Further benchmarking of the dataset (21) reveals that the majority of eluted peptides are within expected binding ranges, but a large fraction of binders are not identified by the elution of experiments (Table 1B). This is not unexpected and is likely reflective of the impact of protein expression/abundance also shaping it; in concert with a binding capacity of the actual peptides, the repertoire of natural ligands bound to MHC. A compensatory relation between binding and expression was indeed noted by Abelin and coworkers, who states. “This revealed a multiplicative relationship between expression and affinity, in which a 10-fold increase in expression could approximately compensate for a 90% decrease in binding potential” (22).

Generating datasets where for a given model antigen we can address which peptides are experimentally found to bind and isolate as natural ligands in the context of HLA class II molecules should be considered a priority for the general field of benchmarking of binding and elution data.

## How Do Algorithms Perform in Predicting The “Other” Variable?

As mentioned above, it is intuitively expected that binding data might be most effective in training to predict binding capacity, but not necessarily eluted ligands. Likewise, training with eluted ligand might be expected to be the most effective to predict eluted ligands but not necessarily HLA binding *per se*. This point was formally addressed by Garde et al. (15). The authors expanded the NNalign approach by adding a second output neuron, and training is performed on both data binding and eluted data simultaneously. The resulting model is able to predict binding affinity value and the likelihood of peptide being an eluted ligand. This study demonstrated that training in class II eluted data increases the accuracy of predicting eluted ligands, but not to predict binding, and that vice versa training in binding data increases the accuracy of predicting binding data, but not to eluted data. In conclusion, these data reiterate that caution must be exercised when algorithms generated to predict a certain variable are used to predict outcomes linked to a different, albeit related, variable. It further sets the stage for the next level of benchmarking, namely, how do HLA class II binding and eluted data and predictions perform when used to predict HLA class II-restricted T-cell immunogenicity?

## Major Histocompatibility Complex Binding Affinity Data as a Predictor of Immunogenicity

In the case of HLA class I, it was originally reported that ~80% of epitopes bind with *K*_d_ < 500 nM (23). The more recent analysis confirmed this observation, supporting this historic threshold (24). It was further found that different alleles are associated with different affinity distributions (24), leading to the recommendation that allele-specific thresholds are preferred when class I binding predictions are used to predict immunogenicity.

**Figure 2:**
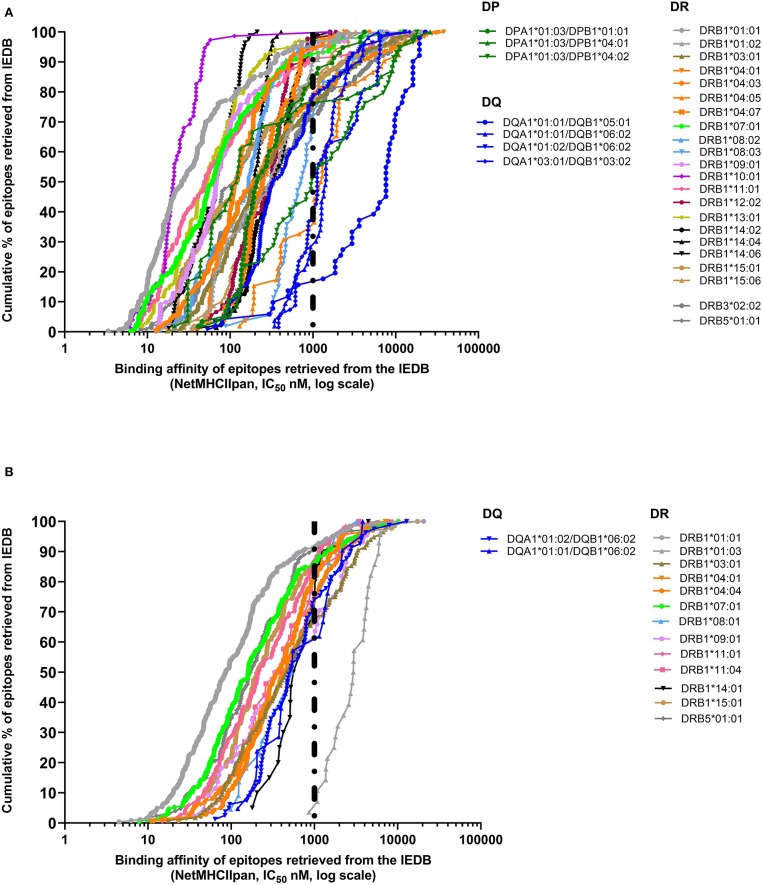
Extrapolation of binding affinity threshold for human leukocyte antigen (HLA) class II prediction. Binding affinity retrieved by NetMHCIIpan by plotting IC_50_ predicted values for each HLA class II allele on the basis of the cumulative percentage of epitopes derived from the Immune Epitope Database and Analysis Resource (IEDB) **(A)** or based only on tetramer data available in IEDB **(B)**.

In the case of class II, a 1,000-nM threshold was suggested, but not extensively validated over large datasets. To address this point, we generated curves capturing percent of epitopes retrieved from the IEDB restricted by different HLA class II molecules, or we generated a higher quality of data, restricting the data considered to be those associated with positive tetramer assays. The results shown in Figure 2A demonstrate that when alleles for at least 50 epitopes have been described with defined restriction, 83.3% epitopes bind at <1,000 nM (3,579 out of 4,297 epitope/allele combinations). As noted in the case of HLA class I, a significant spread exists from one allele to the next. Similarly, when only tetramer data are considered, we plotted data from 15 alleles with at least 20 epitopes (Figure 2B). We found that 80.1% epitopes bind at the <1,000-nM threshold (1,353 out of 1,690 epitope/allele combinations). Table 2 shows the affinity at which 50 and 90% of epitopes are retrieved for each of the HLA alleles described in Figure 2. It is noted that the DRB1^*^01:03 allele has only one epitope at the <1,000-nM level and appears to be an outlier. Whether this reflects a problem with the dataset, or rather the algorithm, or a peculiarity of this rather infrequent allele remains to be investigated.

Rigorous benchmarking of epitopes in a single well-defined system where the epitopes are mapped to different HLA class II molecules is not currently available. The above-referenced study of Croft et al. (19), in addition to studying eluted peptides and measuring binding affinities, also measured the epitopes recognized following VACV infection, also in the context of previously detailed immunogenicity studies (25). The benchmarking study of Paul et al. (12) provides a benchmarking analysis of these data. It was found that the top 1–2% of binding predictions captured 90% of the epitopes or of the total response and that the top 0.03–0.04% of the predicted binders accounted for 50% of the total epitopes and response. The analysis, however, also further underlined how binding predictions are very sensitive predictors but are associated with relatively low specificity. In other words, in the case of murine class I, when 90% of the epitopes are binders, only about 1% of the binders are epitopes. In conclusion, the lack of comprehensive benchmarking of binding prediction and HLA class II-restricted actual immunogenicity is a major knowledge gap, and generating suitable datasets should be considered a priority.

## How Effective is Ligand Elution as a Predictor of Immunogenicity?

Few studies have benchmarked how effectively eluted ligand data can be used in terms of prediction of HLA class II immunogenicity. A study by Mutschlechner et al. (26) compared elution data and T-cell immunogenicity in a case of patients allergic to the known major birch pollen allergens. These authors found that, in general, elution data overlapped with immunogenicity data but missed one of two major T-cell immunogenic sites (around positions 77–93 of the Bet v1 protein).

It is reasonable to assume that all “true” HLA class II epitopes are naturally processed, but it is unclear how many are detected vs. missed given the limits of sensitivity of the assays. High abundance can compensate for low MHC affinity, but it is unknown how immunogenic these types of ligands are. Conversely, a low abundance of ligand that binds with high affinity may be less easily detected but more strongly immunogenic.

## What is the Relative Value of Binding vs. Elution Predictions to Predict Immunogenicity?

As mentioned above, Nielsen, Jurtz, Garde, and associates developed a methodology where binding data, elution data, or both can be used to train NNs, and they generated as output the likelihood that a given sequence will be an HLA class II binder or an eluted ligand. The question that is key in light of application to T-cell immunogenicity is which training is optimal for T-cell epitope predictions. And which output is optimal? The results of this analysis have been recently published (15) and show that training in both ligand and binding datasets is the most effective and that the optimal output is the prediction of eluted ligands. These results have been confirmed by three independent studies (27–29).

Although a formal benchmarking for HLA class II molecules in a controlled experimental system is to date lacking, these results are in strong agreement with the results of the murine class I study of Tscharke in the VACV system (19). In that study, it was found that of a total of 82 epitopes, 60 were both found to be binders in actual binding assays and also experimentally identified as eluted ligands (Table 3) (21). Fifteen epitopes were binders not identified as eluted ligands, whereas five eluted ligands were not experimentally found to be 500-nM binders. However, only two of the peptides experimentally determined to be epitopes were found to be neither binders nor eluted ligands. These data provide compelling evidence that a combination of both predicted binding and elution data should be considered for the purpose of epitope identification.

**Table 3:** Benchmarking summary of HLA class I molecules in the murine VACV system.

		**Predicted binder or not**		
		**Binder**	**Non-binder**	**Total**
Eluted or not	Eluted	60	5	65
	Non-eluted	15	2	17
	Total	75	7	82

*HLA, human leukocyte antigen; VACV, vaccinia virus*.

## The Impact of Human Leukocyte Antigen Polymorphism on Binding vs. Immunogenicity Predictions

HLA polymorphism is an important issue to be considered in evaluating the performance of HLA binding or eluted ligand predictions as a predictor of immunogenicity. HLA class II predictions are by definition allele-specific. However, in real-life drug immunogenicity scenarios, this has to be reconciled with the fact that HLA class II molecules are remarkably polymorphic, encoded by seven different loci, and represented by thousands of different allelic variants.

At the level of individual patients, each human subject is typically heterozygote at four different HLA class II loci (DRB1, DRB3/4/5, DP, and DQ) and therefore expresses up to eight or more different HLA class II variants; this is because of the so-called heterozygous pairing of DP and DQ where both alpha and beta subunits are polymorphic and can form *trans* and *cis* pairings leading to an estimate of about 12 different molecules. And a patient population expresses hundreds of different variants, each represented in different frequencies, which also vary significantly across different ethnicities.

Human immunogenicity and clinical trials rarely determine the specific HLA class II molecule restricting the response, as this is considerably more complex and less clear-cut than in the case of HLA class I. As a result, actionable predictive strategies to target, not alleles, but individuals and populations are required.

Our group has defined a subset of 26 different DRB1, DRB3/4/5, DP, and DQ allelic variants (30) that afford 94.5% global coverage of general human populations. We have used promiscuity indexes (that is, predicting peptides binding to a majority of the most common alleles) as a way to identify peptides that correspond to the most dominant, most immunogenic peptides observed in real-life patient populations (30).

This approach was further optimized, utilizing datasets derived from peptide sets spanning entire proteins associated with measured immune responses in exposed humans to examine a) how many and b) which specific HLA class II variant predictions would be most effective, when combined, to predict immunogenicity in human populations. It was found that optimal results were found with a set of just seven variants, representative of common and dominant class II motif types (31).

## Predicting Immunogenicity *in vivo* in Human Populations

The performance of the “seven-allele method” in predicting immunogenicity in patient populations was evaluated in a subsequent study (32). In the same study, we also considered an agnostic approach, where we used T-cell recognition data to directly train predictive algorithms. For this purpose, we used in-house data and IEDB-derived tetramer as training sets. The performance was evaluated using results from 57 different studies from other laboratories, which used overlapping peptides and exposed populations that contained 530 non-redundant dominant epitopes and 1,758 non-epitopes.

We observed that either the HLA class II binding predictions (seven alleles) or the T-cell immunogenicity tools were associated with overall AUC values of 0.7. Using the two methods in combination afforded modest gains, with AUC of 0.725. The relatively low overall AUC values should not be surprising, given the fact that what is predicted here is not an outcome linked to a given HLA but a population outcome, where the composition of the responding population is unknown and the restricting HLA molecules associated with each epitope are not determined.

## Predicting Immunogenicity *in vivo* by *in vivo* Immunogenicity Assays

*In vitro* assays utilizing cells from naïve, non-exposed donors offer an obvious alternative to bioinformatic predictions. Primary immunogenicity can be measured *in vitro* by a variety of methods. These include immunizing with whole antigen or peptides, using dendritic cells or peripheral blood mononuclear cells (PBMCs) as APC, usually after a period of *in vitro* culture, followed by read-out assays that include proliferation, enzyme-linked immunosorbent spot (ELISPOT), and intracellular cytokine staining (ICS).

Despite their widespread use, benchmarking the performance of these assays as a predictor of *in vivo* immunogenicity is lacking. Rigorous benchmarking studies are required to establish whether these methods do actually predict *in vivo* immunogenicity and which method is most effective. Questions to be addressed include whether memory responses are detected in drug-treated subjects and whether HLA type predicts which subjects will develop memory T-cell responses. It is further unclear to what extent HLA binding, peptide elution, or *in vitro* immunogenicity assays or predictions actually predict which subjects will develop memory T-cell responses. Finally, benchmarking should address at the population level whether binding, elution, or immunogenicity assays or predictions actually identify which epitopes are dominant in *ex vivo* scenarios, with obvious implications for strategies aimed at protein de-immunization by removing T-cell immunogenic epitopes.

## Conclusions

### Do T-Cell Responses Correlate With ADA?

Surprisingly, this is still a very open question that rigorous benchmarking studies can help answer. This will require a global assessment of drug-specific memory T cells in drug-exposed individuals. We believe that the paper makes a clear and desperate plea for the need to generate more data and for honest and objective benchmarking, which are a necessary requisite for moving the field forward. Do the magnitude and/or specificity of memory T-cell responses correlate with ADA titers and/or neutralizing activity? Does immunogenicity (predicted or measured or in non-exposed subjects) predict immunogenicity in exposed subjects? Are the same epitopes recognized as dominant in ADA+ and naïve subjects (with obvious implications for de-immunization) (33)?

It should be emphasized that this review does not address other variables that are appreciated to impact ADA and T-cell immunogenicity, such as induction of T-cell tolerance, self-similarity, protein-drug dosing and schedule, aggregation state, and general immune responsiveness of the drug recipient. In particular, the methods available to the scientific community are trained and derived for the most part on the basis of “strong” infectious diseases and allergy-derived epitopes (with a growing representation of autoimmune and cancer-derived epitopes). In the context of drug immunogenicity and design, it is possible that epitope prediction thresholds might need to be adjusted. This issue can be objectively addressed only when a sufficient amount of epitope data from protein drugs will be accumulated and made public. Ideally, these data could also be utilized to develop algorithms specific to the prediction of drug immunogenicity.

Answering these questions will ultimately require the coming together of bioinformaticians, cellular immunologists, and clinical scientists, applying rigorous and transparent methodologies and datasets. And ultimately, it will require prospective evaluations of immunogenicity including *in vitro* immunogenicity assay pre-exposure, HLA typing, and post-exposure immunogenicity and ADA measures to generate the datasets in which benchmarking can be applied. Ultimately, how can we predict immunogenicity outcome if all we do is run predictions and not test them in a prospective fashion if the immunogenicity assays predicted immunogenicity and ADA outcomes?

## Author Contributions

SP performed specific database queries, generated tables, and wrote the manuscript. AG generated the figures and wrote the manuscript. BP critically reviewed the review. AS conceived, wrote, and critically reviewed the review.

### Conflict of Interest

The authors declare that the research was conducted in the absence of any commercial or financial relationships that could be construed as a potential conflict of interest.
